# Total Syntheses of (±)-Gusanlung A, (±)-Gusanlung D and 8-Oxyberberrubine and the Uncertainty Concerning the Structures of (-)-Gusanlung A, (-)-Gusanlung D and 8-Oxyberberrubine

**DOI:** 10.3390/molecules14020726

**Published:** 2009-02-12

**Authors:** Surachai Nimgirawath, Phansuang Udomputtimekakul, Thitima Apornpisarn, Asawin Wanbanjob, Thongchai Taechowisan

**Affiliations:** 1 Department of Chemistry, Faculty of Science, Silpakorn University, Nakorn Pathom 73000, Thailand; E-mails: Phansuang@yahoo.com (P.U.), winsusc@gmail.com (T.A.), thitima_noon@hotmail.com (T.A.); 2 Department of Microbiology, Faculty of Science, Silpakorn University, Nakorn Pathom 73000, Thailand; E-mail: tthongch@su.ac.th (T.T.)

**Keywords:** Alkaloid, Protoberberine, Isoquinoline, Synthesis, Antimicrobial activity.

## Abstract

(±)-Gusanlung A, 8-oxyberberrubine and (±)-gusanlung D have been synthesized by radical cyclisation of the corresponding 2-aroyl-1-methylenetetra- hydroisoquinolines. The ^1^H and ^13^C spectra of (-)-gusanlung D were found to be different from those of synthetic (±)-gusanlung D. Careful analyses of the ^13^C spectra of (–)-gusanlung A and natural 8-oxyberberrubine also cast doubt on the correctness of the structures previously assigned to these two compounds. (±)-Gusanlung A and (±)-gusanlung D were inactive against *Staphylococcus aureus* ATCC25932, *Escherichia coli* ATCC10536 and *Candida albicans* ATCC90028.

## Introduction

(–)-Gusanlung D, isolated from *Acangelisia gusanlung* H. S. Lo (Menispermaceae), is the first natural 8-oxotetrahydroprotoberberine alkaloid with an unoxygenated ring D [1]. Based on spectral data analysis, structure **1** was proposed for (–)-gusanlung D. Prior to the isolation of (–)-gusanlung D, Kessar *et al*. synthesized in 1992 a compound which is essentially (±)-gusanlung D [2]. However, a close comparison of the ^1^H-NMR data of (±)-gusanlung D with those reported for (–)-gusanlung D revealed significant differences. In 2003 Reimann, Grasberger and Polborn reported another synthesis of (±)-gusanlung D [3]; in this case the ^13^C-NMR spectral data were found to show significant differences to those reported for (–)-gusanlung D. Subsequently, an unsymmetric synthesis of (–)-gusanlung D was achieved by Chrzanowska, Dreas and Razwadowska in 2004 [4]. Comparison of the ^1^H- and ^13^C-NMR data of synthetic (–)-gusanlung D with those of natural (–)-gusanlung D also showed significant differences. Finally, Chang and Chang reported a total synthesis of (±)-gusanlung D [5], whose spectral data were said to agree with those in references [1,2,3,4]. This last conclusion added further confusion to the matter since, if the spectral data of (±)-gusanlung D [5] are in good agreement with those reported for (±)-gusanlung D [2,3] and synthetic (–)-gusanlung D [4], they cannot also be consistent with those reported for natural (–)-gusanlung D [1]. In view of these discrepancies in the ^1^H- and ^13^C-NMR data of natural (–)-gusanlung D [1] and the synthetic alkaloids, it was therefore highly desirable to perform another independent synthesis of (±)-gusanlung D to shed further light on the possible structure of (–)-gusanlung D.

**Figure 1:**
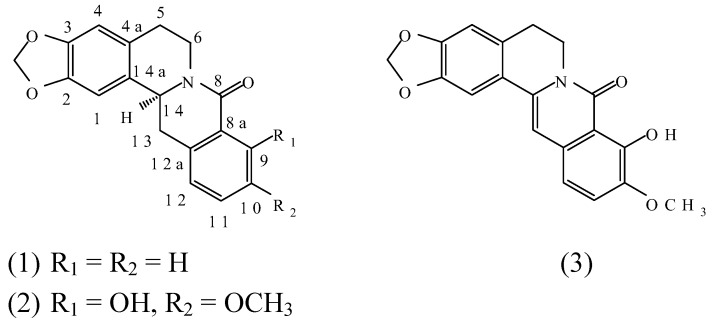
Structures of (–)-gusanlung D (**1**), (–)-gusanlung A (**2**) and 8-oxyberberrubine (**3**).

Furthermore, two new related alkaloids: (–)-gusalung A [**6**] and 8-oxyberberrubine [**1**], for which structures **2** and **3** were proposed based on spectral analysis, were isolated from *Acangelisia gusanlung* H. S. Lo. In view of the uncertainty regarding the correct structure of (–)-gusanlung D (**1**), it was therefore highly desirable to also confirm the correctness of the structures proposed for (–)-gusanlung A (**2**) and 8-oxyberberrubine (**3**) by total syntheses.

## Results and Discussion

### Syntheses of (±)-gusanlung A (**2**) and 8-oxyberberrubine (**3**)

**Scheme 1:**
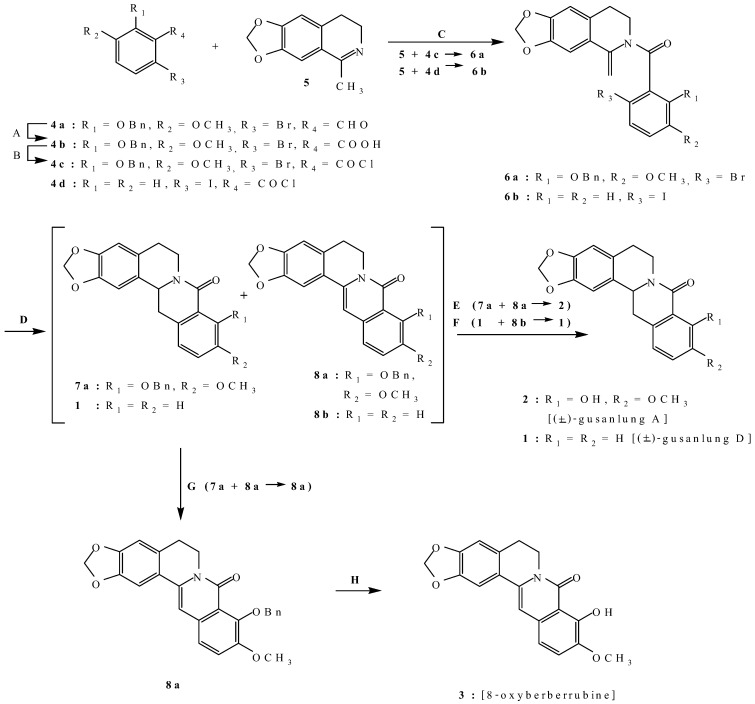
Synthetic routes to (±)-gusanlung A (**1**), (±)-gusanlung D (**2**), and 8-oxyberberrubine (**3**).

The synthesis of (±)-gusanlung A (**2**) was based on the radical-initiated cyclization of 2-(2'-benzyloxy-6'-bromo-3'-methoxybenzoyl)-1-methylene-6,7-methylenedioxy-1,2,3,4-tetrahydroisoqui- noline (**6a**), as outlined in Scheme 1, with subsequent catalytic hydrogenolysis of the benzyl protecting group. 

*Reagents and Conditions* A) NaClO_2_, sulphamic acid/ *tert*-butanol-H_2_O; B) SOCl_2_/ benzene; C) Et_3_N/ dry benzene; D) Bu_3_SnH, AIBN/ dry benzene; E) H_2_, Pd/C/ ethanol; F) hydrazine, Pd/C/ ethyl acetate-ethanol; G) I_2_/ dioxane; H) conc. HCl/ ethanol.

Thus, oxidation of 2-benzyloxy-6-bromo-3-methoxybenzaldehyde (**4a**) [**7**] with sodium chlorite gave 2-benzyloxy-6-bromo-3-methoxybenzoic acid (**4b**), whose acid chloride (**4c**) was then reacted with 6,7-methylenedioxy-1-methyl-3,4-dihydroisoquinoline (**5**) [**8**] in the presence of triethylamine to give thee moderately stable compound **6a**. Treatment of **6a** with tributyltin hydride in the presence of a catalytic amount of 2,2'-azobis(isobutyronitrile) gave a 31.3% yield of a mixture of (±)-9-benzylgusanlung A (**7a**) and 9-benzyl-8-oxyberberrubine (**8a**) in a ratio of 78:22 according to ^1^H-NMR analysis. Catalytic hydrogenolysis of the mixture of **7a** and **8a** to remove the benzyl protecting group also resulted in the concurrent hydrogenation of the C-C double bond to give pure (±)-gusanlung A (**2**). On the other hand, oxidation of the mixture of **7a** and **8a** with iodine gave 9-benzyl-8-oxyberberrubine (**8a**), whose benzyl protecting group was removed by acid treatment to give 8-oxy-berberrubine (**3**).

**Figure 2:**
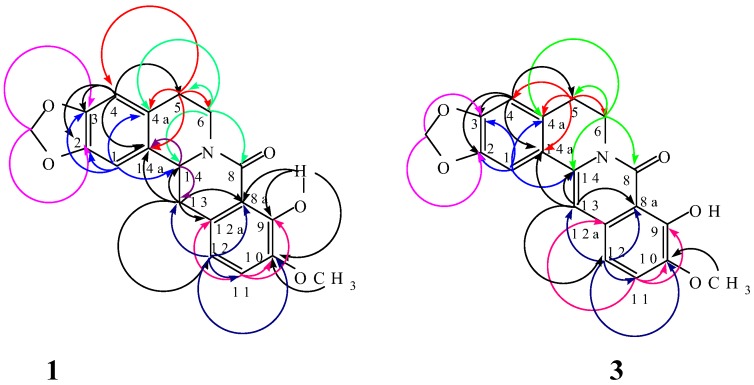
HMBC correlations of (±)-gusanlung A (**1**) and 8-oxyberberrubine (**3**).

The ^1^H-NMR data of synthetic (±)-gusanlung A (**2**) were in reasonably good agreement with those reported for natural (–)-gusanlung A (**2**). However, a number of carbons in the ^13^C-NMR spectrum of natural (–)-gusanlung A (**2**) were found to have quite different chemical shifts from the corresponding carbons in the spectrum of (±)-gusanlung A (**2**). We therefore carried out ^1^H-^1^H-COSY, HMQC and HMBC experiments to allow complete assignments of chemical shifts of (±)-gusanlung A (**2**). Details of the HMBC correlations are shown in Figure 2 and Table 4. The ^1^H-NMR spectral data of natural 8-oxyberberrubine (**3**) were found to be in good agreement with those of synthetic 8-oxyberberrubine (**3**). However, from HMBC correlation experiment, it was possible to establish that the chemical shifts of H-1 and H-13 previously assigned should be interchanged. On the other hand, the ^13^C spectrum of natural 8-oxyberberrubine (**3**) had a number of features which were quite different from those of synthetic 8-oxyberberrubine (**3**). These differences were highlighted and the HMBC correlations were shown in Figure 2 and Table 5. In summary, it can be concluded that while the ^1^H-NMR analysis lent good support to the structures proposed for (–)-gusanlung A (**2**) and 8-oxyberberrubine (**3**), in view of the discrepancies in a number of carbon chemical shifts in the ^13^C-NMR spectra of (-)-gusanlung A (**2**) *versus* those of (±)-gusanlung A (**2**) on the one hand, and natural 8-oxyberberrubine (**3**) *versus* synthetic 8-oxyberberrubine (**3**) on the other, no definite conclusions can be drawn at this time concerning the correctness of the structures previously assigned to (–)-gusanlung A (**2**) and 8-oxyberberrubine (**3**).

### Synthesis of (±)-gusanlung D

The synthesis of (±)-gusanlung D (**1**) was uneventful. Thus, 2-iodobenzoyl chloride (**4d**) was reacted with **5** [**8**] in the presence of triethylamine to give the highly unstable 2-(2'-iodobenzoyl)-1-methylene-6,7-methylenedioxy-1,2,3,4-tetrahydroisoquinoline (**6b**). Treatment of **6b** with tributyltin hydride in presence of a catalytic amount of 2,2'-azobis(isobutyronitrile) gave a 39.0% yield of a mixture of **1** and **8b** in a ratio of 87:23 from ^1^H-NMR analysis. Treatment of the mixture with hydrazine and palladium/charcoal gave (±)-gusanlung D (**1**), whose ^1^H- and ^13^C-NMR data were in good agreement with those of (±)-gusanlung D (**1**) and (–)-gusanlung D obtained from previous syntheses [2,3,4] but differed significantly from those of natural (–)-gusanlung D [1]. The structure previously assigned to (–)-gusanlung D [1] therefore remains uncertain.

**Table 1:** Comparison of ^1^H-NMR spectral data between natural (-)-gusanlung D [**1**], synthetic (-)-gusanlung D [**4**] and synthetic (±)-gusanlung D [**2**] and [**this work**].

(position)	(–)-gusanlung D CDCl_3_ [**1**] m.p. 250-251 °C	(–)-gusanlung DCDCl_3_ [**4**] m.p. 195-197 °C	(±)-gusanlung DCDCl_3_ [**2**] m.p. 175-177 °C	(±)-gusanlung D CDCl_3_ [**this work**] m.p. 175-176 °C
	^1^**H**	^1^**H**	^1^**H**	^1^**H**
1	**7.35** (s)	**6.71** (s)	**6.76** (d)	**6.72** (s)
4	**6.80** (s)	**6.67** (s)	**6.76** (d)	**6.67** (s)
5α	2.70-3.40 (m)	2.7-2.8 (m)	2.83-3.35 (m)	2.70-2.82 (m)
5β	2.70-3.40 (m)	2.82-3.02 (m)	2.83-3.35 (m)	2.87-3.07 (m)
6α	2.70-3.40 (m)	2.82-3.02 (m)	2.83-3.35 (m)	2.87-3.07 (m)
6β	4.8 (m)	4.93-4.99 (m)	4.7-5.1 (m)	4.88-4.99 (m)
9	**8.07** (d, 8.0)	**8.13** (d, 7.4)	**8.1-8.37** (m)	**8.13** (dd, 7.6, 1.4)
10	7.29-7.41 (m)	7.34-7.40 (m)	7.25-7.65 (m)	7.39 (br t, 7.4)
11	7.29-7.41 (m)	7.41-7.49 (m)	7.25-7.65 (m)	7.46 (dt, 7.4, 1.5)
12	7.29-7.41 (m)	7.24 (d, 7.4)	7.25-7.65 (m)	7.22-7.29 (m)
13α	2.70-3.40 (m)	2.82-3.02 (m)	2.83-3.35 (m)	2.87-3.07 (m)
13β	2.70-3.40 (m)	3.18 (dd, 15.3, 3.7)	2.83-3.35 (m)	3.18 (dd, 15.7, 3.7)
14	**3.95** (m)	**4.83** (dd, 13.3, 3.7)	**4.7**-**5.1** (m)	**4.84** (dd, 13.3, 3.7)
OCH_2_O	**6.20, 6.06** (s)	**5.96 (s)**	**5.93** (s)	**5.96 (s)**

**Table 2:** Comparison of ^13^C-NMR spectral data between natural (-)-gusanlung D [**1**], synthetic (-)-gusanlung D [**4**] and synthetic (±)-gusanlung D [**3**] and [**this work**].

(position)	(–)-gusanlung DCDCl_3_ [**1**] m.p. 250-251 °C	(–)-gusanlung DCDCl_3_ [**4**] m.p. 195-197 °C	(±)-gusanlung DCDCl_3_ [**3**] m.p. 175-177 °C	(±)-gusanlung DCDCl_3_ [**this work**] m.p. 175-176 °C
	^13^**C**	^13^**C**	^13^**C**	^13^**C**
1	**107.3**	**105.8**	**105.97**	**105.9**
2	**135.0**	**146.5** ^b^	**146.57**	**146.6**^c^
3	147.0	146.7^b^	146.77	146.8^c^
4	**107.5**	**108.6**	**108.81**	**108.7**
4a	126.5	128.8	128.85	128.9
5	29.7	29.6	29.61	29.7
6	**42.0**	**38.7**	**38.49**	**38.8**
8	**162.0**	**164.5**	**158.67**	**164.6**
8a	**117.3**	137.2	137.24	**137.3**
9	128.7^a^	128.6	128.60	128.6
10	**127.9**^a^	**127.3**	**127.37**	127.4^*^
11	**127.1**^a^	**131.8**	**132.33**	131.9^*^
12	126.8^a^	126.8	126.87	126.9
12a	**124.6**	**129.0**	**131.81**	**129.1**
13	**33.5**	**38.1**	**37.78**	**38.1**
14	**49.4**	**55.2**	**55.18**	55.3
14a	**126.5**	**128.5**	**128.55**	**128.6**
OCH_2_O	100.9	101.1	101.00	101.1

^a, b, c,^* assignments may be interchangeable.

**Table 3:** Comparison of ^1^H-NMR spectral data between natural (-)-gusanlung A [**1**] and synthetic (±)-gusanlung A [**this work**].

(position)	(-)-gusanlung A (DMSO-*d*_6_) [**6**] m.p. 260-262 °C	(±)-gusanlung A (DMSO-*d*_6_) [**this work**] m.p. 188-189 °C	(±)-gusanlung A (CDCl_3_) [**this work**] m.p. 188-189 °C
	**^1^H**	**^1^H**	**^1^H**
1	6.96 (s)	7.00 (s)	6.71 (s)
4	6.80 (s)	6.79 (s)	6.66 (s)
5	2.73-2.81 (m)	2.75-2.89 (m)	2.72-2.84 (m)
6α	**2.73-2.81 (m)**	**2.89-3.01 (m)**	2.94-3.40 (m)
6β	**4.71 (m)**	**4.69**-**4.59(m)**	4.80-4.87 (m)
11	6.99 (d, 8.1)	7.09 (d, 8.1)	6.94 (d, 8.1)
12	**6.86 (d, 8.1)**	**6.71 (d, 8.1)**	6.63 (d, 8.1)
13α	**3.13 (dd, 15.3, 3.1)**	**3.36 (dd, 15.2, 3.6)**	3.14 (dd, 15.2, 3.8)
13β	2.62 (dd, 15.3, 13.3)	2.66-2.75 (m)	2.80-2.94 (m)
14	**4.68 (dd, 13.3, 3.1)**	**4.84 (dd, 13.3, 3.4)**	4.80 (dd, 13.6, 3.5)
C_10_-OCH_3_	3.76 (s)	3.78 (s)	3.90 (s)
OCH_2_O	5.98, 5.99 (s)	5.98, 6.00 (s)	5.96 (s)
OH	-	12.88 (s)	12.83 (s)

**Table 4:** Comparison of ^13^C-NMR spectral data between natural (-)-gusanlung A [**6**] and synthetic (-)-gusanlung A [**this work**] and HMBC correlations of (±)-gusanlung A [**this work**].

(position)	(-)-gusanlung A (DMSO-*d*_6_) [**6**] m.p. 260-262 °C	(±)-gusanlung A (DMSO-*d*_6_) [**this work**] m.p. 188-189 °C	(±)-gusanlung A (CDCl_3_) [**this work**] m.p. 188-189 °C	(±)-gusanlung A (DMSO-*d*_6_) [**this work**]	
				HMBC	
	^13^**C**	^13^**C**	^13^**C**	**2J**	**3J**
1	106.1	106.6	105.8	C-2	C-3, 4a, 14
2	145.9^a^	146.7^c^	146.8*	-	-
3	147.7^a^	146.5^c^	146.7*	-	-
4	**107.8**	**108.7**	108.6	C-3	C-2, 5, 14a
4a	129.1^b^	128.3	128.1	-	-
5	29.0	28.9	29.4	C-4a, 6	C-4, 14a
6	**37.8**	**38.5**	38.4	C-5	C-4a, 8, 14
8	**161.4**	**168.4**	168.6	-	-
8a	**122.3^b^**	**111.4**	111.4	-	-
9	149.7^a^	151.4	151.8	-	-
10	145.7^a^	147.2	147.5	-	-
11	**118.9**	**116.7**	115.4	C-10	C-9, 12a
12	**122.1**	**116.9**	116.1	C-11	C-8a, 10, 13
12a	**128.2**^b^	**129.6**	128.7	-	-
13	**37.7**	**35.9**	37.1	C-12a, 14	C-8a, 12, 14a
14	**54.4**	**55.4**	55.7	C-13, 14a	-
14a	**129.3**^b^	**129.1**	128.4	-	-
C_10_-OCH_3_	**60.5**	**56.3**	56.3	-	C-10
OCH_2_O	**100.5**	**101.3**	101.2	-	C-2, 3
OH				C-9	C-8a, 10

^a, b, c,^* assignments may be interchangeable.

**Table 5:** Comparison of ^1^H- and ^13^C-NMR spectral data between natural 8-oxyberberubine (**3**) [**1**], synthetic 8-oxyberberubine (**3**) [**this work**] and HMBC correlations of 8-oxyberberrubine [**this work**].

(position)	natural 8-oxy-berberrubine (**3**) CDCl_3_ [**1**] m.p. 240-241 °C	synthetic 8-oxy-berberrubine (**3**) CDCl_3_ [**this work**] m.p. 238-239 °C	natural 8-oxy-berberrubine (**3**) CDCl_3_ [**1**] m.p. 240-241 °C	synthetic 8-oxy-berberrubine (**3**) CDCl_3_ [**this work**] m.p. 238-239 °C	synthetic 8-oxyberberrubine (**3**) (CDCl_3_) [**this work**]	
					HMBC	
	^1^**H**	^1^**H**	^13^**C**	^13^**C**	**2J**	**3J**
1	**6.83 (s)**	**7.21 (s)**	104.0	104.8	C-2	C-3, 4a, 14
2			**141.6**	147.5*	-	-
3			**146.4**	148.6*	-	-
4	6.72 (s)	6.71 (s)	107.1	108.0	C-3	C-2, 5, 14a
4a			**109.6**	**129.5**	-	-
5	2.91 (t, 7.2)	2.92 (t, 6.1)	28.4	28.4	C-4a, 6	C-4, 14a
6	4.27 (t, 7.2)	4.27 (t, 6.1)	39.1	39.1	C-5	C-4a, 8, 14
8			**164.0**	**165.4**	-	-
8a			**129.9**	**111.0**	-	-
9			149.0	150.3	-	-
10			**147.5**	**144.9**	-	-
11	7.30 (AB q, 8.0)	7.28 (d, 8.5)	**114.9**	**119.1**	C-10	C-9, 12a
12	7.00 (AB q, 8.0)	6.99 (d, 8.5)	**120.0**	**115.3**	C-11	C-8a, 10, 13
12a			**128.9**	**130.5**	-	-
13	**7.21 (s)**	**6.83 (s)**	103.6	103.6	C-14	C-8a, 12, 14a
14			133.6	134.6	-	-
14a			**122.1**	**123.5**	-	-
C_10_-OCH_3_	3.96 (s)	3.97 (s)	56.7	56.7	-	C-10
OCH_2_O	6.02 (s)	6.02 (s)	**100.6**	**101.5**	-	C-2, 3
OH	-	13.14	-	-	-	-

*assignments may be interchangeable.

### Antimicrobial activity

(±)-Gusanlung D (**1**) and (±)-gusanlung A (**2**) at the concentration value 256 μg/mL were inactive against *Staphylococcus aureus* ATCC25932, *Escherichia coli* ATCC10536 and *Candida albicans* ATCC90028.

## Conclusions

Based on spectral analysis, there were significant discrepancies between the spectral data of natural (-)-gusanlung D and synthetic (±)-gusanlung D. Hence, the structure previously proposed for (-)-gusanlung D remains doubtful. While the ^1^H spectral data of natural (-)-gusanlung A and 8-oxyberberrubine were in reasonably good agreement with those of synthetic (±)-gusanlung A and 8-oxyberberrubine, the ^13^C spectral data of natural (-)-gusanlung A and 8-oxyberberrubine were not entirely in good agreement with those of synthetic (±)-gusanlung A and 8-oxyberberrubine. The structures previously proposed for natural (-)-gusanlung A and 8-oxyberberrubine must therefore be treated with caution.

## Experimental

### General

Melting points were determined on a SMP 2 Stuart Scientific melting point apparatus and are uncorrected. Infrared spectra were recorded on CH_2_Cl_2_-films with a Perkin Elmer Spectrum GX FT-IR spectrophotometer. Ultraviolet spectra were recorded on methanol solutions with a Perkin Elmer Lambda 35 UV-VIS spectrophotometer. ^1^H- and ^13^C-NMR spectra were recorded on (D) chloroform solutions at 300 MHz for ^1^H and 75 MHz for ^13^C with a Bruker AVANCE 300 spectrometer. Tetramethylsilane was used as the internal standard. MS spectra were recorded on a POLARIS Q mass spectrometer. 

*2-Benzyloxy-6-bromo-3-methoxybenzoic acid* (**4b**). A solution of sodium chlorite (0.36 g, 3.6 mmol) in H_2_O (5 mL) was added to a solution of 2-benzyloxy-6-bromo-3-methoxybenzaldehyde (**4**a) [7] (1.0 g, 3.1 mmol) and sulfamic acid (0.5 g) in *tert*-butanol (10 mL) and H_2_O (3 mL). The solution was stirred for 1 h. The mixture was shaken with ethyl acetate (20 mL) and the ethyl acetate layer was extracted with 5% sodium carbonate (3 × 20 mL). The aqueous layer was then acidified with concentrated hydrochloric acid and extracted with chloroform (3 × 20 mL). The chloroform layer was dried over anhydrous sodium sulfate. Removal of the solvent under vacuum gave a solid which was recrystallized from benzene-hexane to give **4b** as pale white crystals (0.8 g, 76.2%), m.p. 112-115 °C; ^1^H-NMR: δ 7.47-7.42 (2H, m, Ph-H); 7.38-7.25 (4H, m, Ph-H × 3 and Ar-H); 6.88, (1H, d, *J* = 8.9 Hz, Ar-H); 5.10 (2H, s, CH_2_Ph); 3.89 (3H, s, OCH_3_). ^13^C-NMR: δ 171.0 (C), 152.2 (C), 145.9 (C), 136.7 (C), 130.6 (C), 128.4 (CH), 128.3 (CH), 128.2 (CH), 114.9 (CH), 108.7 (C), 76.0 (CH_2_), 56.2 (OCH_3_).

*2-(2'-Benzyloxy-6'-bromo-3'-methoxybenzoyl)-1-methylene-6,7-methylenedioxy-1,2,3,4-tetrahydro-isoquinoline* (**6a**). A solution of acid **4b** (3.6 g, 10.0 mmol) and thionyl chloride (3.9 g, 32.8 mmol) in benzene (20 mL) was refluxed for 1 h. The solvent and excess thionyl chloride were removed under vacuum to give acid chloride **4c** as a yellow oil (3.7 g, 94.9%) which was used in the next step without further purification. A solution of acid chloride **4c** (1.9 g, 5.3 mmol) in dry benzene (20 mL) was added dropwise over 10 min. to a solution of isoquinoline **5** [**8**] (1.0 g, 5.3 mmol) and triethylamine (1.0 g) in dry benzene (20 mL), then the mixture was refluxed for 2 h. On cooling, the precipitated triethylamine hydrochloride was filtered off. The filtrate was evaporated under vacuum to give enamide **6a** as a yellow oil (2.6 g, 84.4%) which was unstable and decomposed on standing. It was immediately used in the next step without further purification. ^1^H-NMR: δ 7.38-7.23 (5H, m, Ph-H), 7.18(1H, d, *J* = 8.8 Hz, H-5'), 6.89 (1H, s, H-8), 6.75 (1H, d, *J* = 8.8 Hz, H-4'), 6.41 (1H, s, H-5), 5.90 (2H, AB q, *J* = 1.3 Hz, OCH_2_O), 5.14 (1H, d, *J* = 1.3 Hz, =CH_2_), 5.00 (2H, AB q, *J* = 10.8 Hz, CH_2_Ph), 4.81 (1H, d, *J* = 1.3 Hz, =CH_2_), 4.13-4.02, 3.57-3.50 (2H, 2 m, CH_2_-3), 3.80 (3H, s, OCH_3_), 2.90-2.59 (2H, m, CH_2_-4); ^13^C-NMR: δ 165.0 (C), 152.1 (C), 147.8 (C), 146.5 (C), 145.3 (C), 141.4 (C), 137.4 (C), 134.3 (C), 129.0 (C), 128.4 (CH), 128.1 (CH), 127.9 (CH), 127.7 (CH), 125.1 (C), 113.4 (CH), 110.0 (C), 108.4 (CH), 104.4 (CH_2_), 103.8 (CH), 101.1 (CH_2_), 75.4 (CH_2_), 55.9 (OCH_3_), 41.6 (CH_2_), 28.8 (CH_2_).

*(±)-Gusanlung A* (**1**) *and* *9-benzyl*-*8-oxyberberrubine* (**8a**). A solution of enamide **6a** (2.7 g, 5.3 mmol), tributyltin hydride (3.4 g, 11.7 mmol) and 2,2'-azobis(isobutyronitrile) (0.2 g, 0.7 mmol) in dry benzene (50 mL) was refluxed with stirring for 3 h., then the solvent was removed under vacuum. The residue was washed with hexane (4 × 15 mL) and dissolved in chloroform (30 mL). The chloroform layer was washed with brine, then dried over anhydrous sodium sulfate. The solvent was removed under vacuum to give a yellow solid which was recrystallized from ethanol to give a 31.3% yield of a mixture of (±)-9-benzylgusanlung A (**7a**) and 9-benzyl-8-oxyberberrubine (**8a**) in a ratio of 78:22 from ^1^H-NMR analysis.

A solution of the mixture of **8a** and **7a** (303.7 mg, 0.7 mmol) in ethanol (50 mL) was hydrogenated over 10% Pd/C (30.4 mg) at atmospheric pressure for 48 h. The catalyst was fittered off and the solvent was removed under vacuum to give a crude yellow solid. Recrystallization of the crude solid from ethanol gave (±)-gusanlung A (**2**) as a pale yellow soild (82.4 mg, 34.3%), m.p. 188-189 °C; UV (MeOH) λ_max_ nm (log ε): 219 (4.54), 271sh (3.87), 281 (3.98), 308 (4.16), 319 (4.15); IR ν_max_ (film): 3737, 3650, 3585, 2919, 2852, 1748, 1634, 1615, 1581, 1542, 1506, 1488, 1456, 1386, 1356, 1336, 1315, 1262, 1239, 1154, 1084, 1069, 1037, 1001, 933, 858, 804, 792, 728 cm^-1^; MS (EI) m/z (%): 339 (M^+^, 55), 176 (100). ^1^H-NMR see Table 3, ^13^C-NMR and HMBC see Table 4.

A solution of iodine (4.6 g, 18.3 mmol) in dioxane (100 mL) was added dropwise over 30 min. to a refluxing solution of the mixture of **7a** and **8a** (1.3 g, 3.0 mmol) and sodium acetate (1.5 g) in dioxane (50 mL), then the mixture was refluxed for 6 h. On cooling, the sodium acetate was filtered off and the precipitate was washed with chloroform (100 mL). The chloroform layer was washed with 5% NaHSO_3_ (100 mL), dilute NH_3_ (30 mL), H_2_O (100 mL) then dried over anh. Na_2_SO_4_. Removal of the solvent under vacuum gave a red solid which was recrystallized with ethanol to give 9-benzyl-8-oxyberberrubine (**8a**) as red crystals (0.6 g, 50.0%), m.p. 190-192 °C. UV (MeOH) λ_max_ nm (log ε): 206sh (4.62), 224 (6.31), 255sh (5.78), 312 (5.76), 342 (6.03), 369 (5.86), 387 (5.71); IR ν_max_ (film): 2938, 2898, 2841, 1651, 1619, 1599, 1494, 1484, 1386, 1372, 1317, 1277, 1225, 1176, 1100, 1083, 939, 871, 834, 777, 734 cm^-1^; ^1^H-NMR: δ 7.73-7.68 (2H, m, Ph-H); 7.43-7.32 (3H, m, Ph-H); 7.32-7.28 (2H, m, H-11 and H-12); 7.22 (1H, s, H-1), 6.72 (1H, s, H-13); 6.70 (1H, s, H-4); 6.00 (2H, s, OCH_2_O); 5.16 (2H, s, CH_2_Ph); 4.31 (2H, t, *J* = 6.1 Hz, CH_2_-6); 3.88 (3H, s, OCH_3_); 2.88 (2H, t, *J* = 6.1 Hz, CH_2_-5); ^13^C-NMR: δ 160.2 (C), 151.7 (C), 148.4 (C), 148.2 (C), 147.3 (C), 138.1 (C), 135.6 (C), 132.4 (C), 130.1 (C), 128.7 (CH), 128.2 (CH), 127.7 (CH), 123.8 (C) , 122.5 (CH), 119.8 (C), 119.1 (CH), 107.9 (CH), 104.7 (CH), 101.4 (CH_2_), 101.3 (CH), 75.7(CH_2_), 56.9 (OCH_3_), 39.5 (CH_2_), 28.7 (CH_2_). 

*8-Oxyberberrubine* (**3**). A solution of **8a** (100.0 mg, 0.2 mmol) in ethanol (30 mL) and conc. HCl (30 mL) was refluxed for 3 h. On cooling, the solution was extracted with chloroform (50 mL). The extract was washed with water (50 mL), then dried over anh. Na_2_SO_4_. Removal of the solvent under vacuum gave a yellow solid which was recrystallized with ethanol to give 8-oxyberberrubine (**3**) as pale yellow crystals (42.2 mg, 53.5%), m.p. 238-239 °C (Lit. [2] m.p. 240-241 °C); UV (MeOH) λ_max_ nm (log ε): 225 (4.44), 258sh (3.99), 270 (3.87), 288 (3.69), 345 (4.16), 369 (4.13); IR ν_max_ (film): 3011, 2893, 2836, 1645, 1594, 1490, 1393, 1320, 1267, 1228, 1181, 1087, 1033, 932, 826, 665 cm^-1^. ^1^H-NMR, ^13^C-NMR and HMBC see Table 5.

*2-(2'-Iodobenzoyl)-1-methylene-6, 7-methylenedioxy-1,2,3,4-tetrahydroisoquinoline* (**6b**). A solution of 2-iodobenzoyl chloride **4d** (1.4 g, 5.4 mmol) in dry benzene (20 mL) was added dropwise over 10 min. to a solution of isoquinoline **5** [8] (1.0 g, 5.3 mmol) and triethylamine (1.0 g) in dry benzene (20 mL), then the mixture was refluxed for 2 h. On cooling, the precipitated triethylamine hydrochloride was filtered off and the filtrate was evaporated under vacuum to give enamide **6b** as a yellow oil (2.2 g, 99.1%) which was unstable and decomposed on standing, so it was immediately used in the next step without further purification. ^1^H-NMR: δ 8.07-6.84 (5H, m, Ar-H); 6.58 (1H, s, Ar-H); 5.92 (2H, s, OCH_2_O); 5.18 (1H, br s, =CH_2_); 4.50 (1H, br s, =CH_2_); 4.12( 2H, br s, CH_2_); 2.95 (2H, br s, CH_2_); ^13^C-NMR: δ 169.0 (C), 161.2 (C), 148.2 (C), 146.8 (C), 142.6 (C), 142.2 (CH), 139.3 (CH), 135.9 (C), 132.5 (C), 129.9 (CH), 128.3 (CH), 125.0 (C), 108.4 (CH), 106.2 (CH_2_), 103.9 (CH), 101.2 (CH_2_), 41.8 (CH_2_), 29.0 (CH_2_).

*(±)-Gusanlung D* (**1**) *and 13,14-didehydrogusanlung D* (**8b**)*.* A solution of enamide **6b** (2.9 g, 10.0 mmol) tributyltin hydride (11.7 g, 40.0 mmol) and 2,2'-azobis(isobutyronitrile) (1.6 g, 10.0 mmol) in dry benzene (50 mL) was refluxed with stirring for 3 h., then the solvent was removed under vacuum. The residue was washed with hexane (4 ×15 mL) and dissolved in chloroform (30 mL). The chloroform layer was washed with brine, then dried over anhydrous sodium sulfate. The solvent was removed under vacuum to give a solid which was recrystallized from ethanol to give a 39.0% yield of a mixture of **1** and **8b** in a ratio of 23:87 from ^1^H-NMR analysis.

A mixture of **1** and **8b** (200.0 mg, 0.7 mmol), Pd/C (300.0 mg), hydrazine (50 mL), ethanol (50 mL) and ethyl acetate (50 mL) was refluxed for 48 h. The Pd/C was filtered and the filtrate extracted with chloroform (80 mL). The extract was washed with 10% HCl (2 × 50 mL), water (50 mL) then dried over anh. Na_2_SO_4_. Removal of the solvent under vacuum gave a yellow solid which was recrystallized with ethanol to give pure (±)-gusanlung D (**1**) as pale yellow crystals (99.4 mg, 49.4%), m.p. 175-176 °C (lit. [**5**] m.p. 175-177 °C). UV (MeOH) λ_max_ nm (log ε): 206 (6.27), 230 (5.78), 253sh (5.42), 290 (5.42), 335 (5.02), 365 (4.77); IR ν_max_ (film): 2922, 1646, 1602, 1576, 1487, 1412, 1362, 1333, 1285, 1241, 1218, 1178, 1141, 1038, 936, 906, 853, 743, 636, 505 cm^-1^. ^1^H-NMR and ^13^C-NMR see Table 1 and Table 2.

#### Minimum inhibitory concentration (MIC)

MIC of (±)-gusanlung A (**2**) and (±)-gusanlung D (**1**) were determined by NCCLS microbroth dilution methods [9]. (±)-Gusanlung A (**2**) and (±)-gusanlung D (**1**) were weighed and dissolved in DMSO to make a solution of concentration 2.56 mg/mL. From this stock solution two-fold serial dilution has been carried out to give a series of solutions from 256 μg/mL to 0.50 μg/mL with culture medium in 96-well microplates (100 μL of total volume). Three different microorganisms were selected *viz.* *Staphytolcoccus aureus* ATCC25932, *Escherichia coli* ATCC10536 and *Candida albicans* ATCC90028. They were subcultured on nutrient broth supplemented with 10% glucose (NBG) (for bacteria) or Sabouraud glucose broth (for yeast) and incubated at 37 °C for 24 h. A final concentration of 1 x 10^5^ cfu/mL of test bacteria or yeast was added to each dilution. The plates were incubated at 37 °C for 48 h. MIC was defined as the lowest concentration of test agent that inhibited bacterial or yeast growth, as indicated by the absence of turbidity. Test agent-free broth containing 5% DMSO was incubated as growth control. 
